# Insecticidal Terpenes From the Essential Oils of *Artemisia nakaii* and Their Inhibitory Effects on Acetylcholinesterase

**DOI:** 10.3389/fpls.2021.720816

**Published:** 2021-08-12

**Authors:** Jiayi Liu, Juan Hua, Bo Qu, Xuanyue Guo, Yangyang Wang, Meini Shao, Shihong Luo

**Affiliations:** ^1^College of Bioscience and Biotechnology, Shenyang Agricultural University, Shenyang, China; ^2^Key Laboratory of Biological Invasions and Global Changes, Shenyang, China

**Keywords:** *Artemisia nakaii*, essential oils, insecticidal effects, terpenes, acetylcholinesterase

## Abstract

Essential oils (EOs) are often the source of insecticidal substances of high efficiency and low toxicity. From gas chromatograph-mass spectrometer, column chromatography, and nuclear magnetic resonance spectra analyses, twenty terpenes were identified from the EOs of *Artemisia nakaii*. These comprised mostly monoterpenes (49.01%) and sesquiterpenes (50.76%). The terpenes at the highest concentrations in the EOs of *A. nakaii* were feropodin (200.46 ± 1.42 μg/ml), (+)-camphor (154.93 ± 9.72 μg/ml), β-selinene (57.73 ± 2.48 μg/ml), and 1,8-cineole (17.99 ± 1.06 μg/ml), calculated using area normalization and external standards. The EOs were tested for biological activity and showed strong fumigant toxicity and significant antifeedant activity against the larvae of *Spodoptera litura*. Furthermore, the monoterpenes 1,8-cineole and (+)-camphor displayed significant fumigant activity against *S. litura*, with LC_50_ values of 7.00 ± 0.85 and 18.16 ± 2.31 μl/L, respectively. Antifeedant activity of the sesquiterpenes feropodin and β-selinene was obvious, with EC_50_ values of 12.23 ± 2.60 and 10.46 ± 0.27 μg/cm^2^, respectively. The EOs and β-selinene were also found to inhibit acetylcholinesterase, with IC_50_ values of 37.75 ± 3.59 and 6.88 ± 0.48 μg/ml, respectively. These results suggest that monoterpenes and sesquiterpenes from the EOs of *A. nakaii* could potentially be applied as a botanical pesticides in the control of *S. litura*.

## Introduction

More than 60% of lepidopteran species are major pests, causing serious damage to agriculture and forests (Lee et al., 2019). *Spodoptera litura* (Lepidoptera: Noctuidae) larvae have voracious and polyphagous eating patterns. The damage of *S. litura* has become very serious in Asia, and this species was considered as a “superpest” (Zhang et al., 2021). It can completely destroy or seriously reduce yields in crops of more than 120 economically important species, including cereal crops, vegetables, and ornamental plants. Large amounts of insecticides have been used to control this species for many decades (Wang et al., 2015). However, widespread abuse of chemical pesticides has led to ecological, environmental, and health hazards, and to increased resistance and resurgence in the insects, as well as residue problems (Telang et al., 2003; Werrie et al., 2021). Therefore, the discovery of new, relatively safe, natural products effective against pest species has become increasingly important.

Plant essential oils (EOs) have a wide spectrum of biological activities and practical applications, and several studies have investigated the insecticidal activities of EOs and their potential usage in the poisoning of agricultural pests (Singh and Pandey, 2018; Yin et al., 2018; Cazella et al., 2019). For instance, EOs from *Eucalyptus* can serve directly as a natural insect repellent and have been commercialized in organic agriculture (Batish et al., 2008). *Artemisia annua* EOs had showed acute toxicity against adults and larvae of *Tribolium castaneum* (Deb and Kumar, 2020). Simultaneously, a few EOs have been reported to demonstrate high toxicity against *S. litura*, including EOs extracted from *Wedelia prostrata*, *Origanum creticum*, and *Satureja hortensis* (Hummelbrunner and Isman, 2001; Isman et al., 2001; Vasantha-Srinivasan et al., 2016; Benelli et al., 2018). Essential oils tend to make superior pesticides because they are hypotoxic to mammals and do not generate dangerous residues in either water or soil, which in other pesticides can cause substantial environmental pollution (Singh and Pandey, 2018; Kundu et al., 2020). The development of plant EOs and the substances in them as pesticides against *S. litura* therefore deserves further research.

Plant EOs are complex mixtures of specialized metabolites. Terpenes, which are the major constituents of plant EOs (Cazella et al., 2019), have been found to be commonly toxic for certain insects (Passreiter et al., 2004; Roh et al., 2020). Linalool, from the EOs extracted from the fruits of *Evodia lenticellata* Huang, exhibits both contact and fumigant toxicity against many species of insects, including *Lasioderma serricorne*, *T. castaneum*, and *Liposcelis bostrychophila* (Cao et al., 2018). Bornyl acetate has been tested for fumigant activity against adults of *Callosobruchus chinensis* and *Sitophilus oryzae* (Park et al., 2003). Moreover, many terpenes have been reported to be toxic to *S. litura*. Pogostone is abundant in the EOs extracted from *Pogostemon cablin* and shows antifeedant and larvicidal activities against *S. litura* (Huang et al., 2014). The monoterpenoid thymol exhibits significant toxicity against *S. litura* and can be obtained from the EOs from a number of plant species, including *Thymus vulgaris* (Marchese et al., 2016). Thus, to investigate further insecticidal compounds from plant EOs is a worthwhile and interesting task.

*Artemisia nakaii* Pamp. is a biennial herb in the Asteraceae family, with a mainly Chinese and Korean distribution (Ru, 2018). The plants reach a height of between 30 and 60 cm, and usually grow a taproot. The inflorescence is a turbinate capitulum, flowering and fruiting from August to October in elliptic and obovate achenes. An important distinguishing character of this species is that the shape of the basal leaves is different from that of the cauline leaves. Plants of this species grow vigorously in Liaoning province, China, and in our field studies, we have only seldom observed herbivores feeding on it. Large quantities of volatile components with a pungent smell are rapidly released from the leaves after mechanical injury or touch, and we therefore supposed that leaves contained volatile toxic components worthy of study. The present research confirmed that the EOs of *A. nakaii* exhibit both antifeedant and fumigant toxicity against the larvae of *S. litura*. Moreover, the chemical components of the EOs were elucidated using gas chromatograph-mass spectrometer (GC-MS), chromatographic separation, and nuclear magnetic resonance (NMR) spectroscopy analyses. Bioassays suggested that acetylcholinesterase (AChE) might be a potential target of the insecticidal activity.

## Materials and Methods

### Plant Materials and Field Observations

The *A. nakaii* plant material was collected from the region of Panjing City (E: 121° 46', N: 40° 54') in China in September 2020. Professor Bo Qu identified the samples and collected the voucher specimens (SYNUF021264), which are stored at the College of Bioscience and Biotechnology, Shenyang Agricultural University. A field experiment was also performed in September 2020, to observe insects feeding on *A. nakaii* in its natural state.

### Extraction and Isolation of EOs From *A. nakaii*

The EOs were obtained by the hydro-distillation of 20 *g* of *A. nakaii* aerial parts using a Clevenger-type apparatus for 2 h (Bai et al., 2020). The oil/water mixtures were extracted with hexane, and the hexane layer was washed with anhydrous sodium sulfate, dried, and concentrated. The resulting EOs were then refrigerated in a dark vial until further analysis and experiment. The extraction of EOs was carried out three times to make three replicates.

### GC-MS Analyses

The EOs from *A. nakaii* were analyzed in a Shimadzu GC-MS QP-2020 (Shimadzu, Japan) equipped with an auto-injector AOC-20is. The EOs were segregated on a SH-RXI-17Sil MS column (30 m × 0.25 mm, film thickness 0.25 μm) with helium as the carrier gas, at a flow rate of 1.78 ml/min. Working conditions were as follows: 1 μl of each sample; split mode, split ratio of 10:1; and injector temperature, 250°C. The oven temperature was programmed with an initial oven temperature of 40°C, increased to 80°C at 5°C/min and held for 4 min, then raised to 280°C at 10°C/min, and held for 3 min. The mass detector was operated in electronic impact (EI) mode at 70 eV, installing a scan range of *m/z* 50–500. Ion source, transfer-line, and quadrupole temperatures were set to 230, 250, and 150°C, respectively. A series of n-hydrocarbons (C_8_–C_30_) have been analyzed for calculating of retention indices (RI). Target compounds were identified qualitatively and were quantified using the integration of peak areas, calibration, and comparison with the internal standard (nonane).

### Isolation of Main Constituents of the EOs

About 8 ml of the EOs extracted from *A. nakaii* was collected by hydro-distillation from the plant aerial parts. These EOs were then subjected to chromatography on silica gel columns and eluted with petroleum ether: acetone (v/v, 120:1–1:1) to give eight fractions (fractions 1–8). Fraction 2 (180 mg) was purified using reversed-phase semi-preparative HPLC (87% acetonitrile in water; 3 ml/min; Eclipse XDB-C_18_ column, 5 μm, 9.4 × 250 mm) to yield compound **5** (13 mg, *t*_R_ 39.5 min). Fraction 6 (706 mg) was subjected to chromatography on a sephadex LH-20 column (with acetone as the eluent) and was finally purified with semi-preparative HPLC (61% methanol in water) to yield compound **18** (6 mg, *t*_R_ 33.2 min), compound **19** (9 mg, *t*_R_ 31.2 min), and compound **20** (1 mg, *t*_R_ 33.1 min).

### Spectroscopic Analysis of Purified Compounds

Spectra data of β-selinene (**5**): colorless oils, ^1^H-NMR (600 MHz, CD_3_COCD_3_) *δ*: 4.72 (1H, s, H-14), 4.69 (1H, s, H-14), 4.67 (1H, s, H-13), 4.43 (1H, s, H-13), 2.28 (1H, d, *J* = 12.8 Hz, H-6), 1.99 (2H, dd, *J* = 8.4, 11.8 Hz, 2H-3), 1.84 (1H, d, *J* = 12.2 Hz, H-9), 1.73 (3H, s, CH_3_-12), 1.60 (2H, m, H-5, H-7), 1.52 (3H, m, H-5, H-7, H-8), 1.48 (1H, m, H-8), 1.44 (1H, m, H-2), 1.29 (3H, m, 2H-1, H-2), 0.72 (3H, s, CH_3_-15); ^13^C-NMR (125 MHz, CD_3_COCD_3_) δ: 151.6 (C-4), 151.2 (C-11), 108.7 (C-13), 105.8 (C-14), 50.4 (C-9), 46.6 (C-6), 42.5 (C-1), 41.7 (C-8), 37.4 (C-3), 36.5 (C-10), 30.2 (C-5), 27.5 (C-7), 24.1 (C-2), 21.0 (C-12), 16.6 (C-15).

Spectra data of feropodin (**19**): colorless crystals, ^1^H-NMR (600 MHz, CD_3_COCD_3_) δ: 6.38 (1H, s, H-3), 6.16 (1H, d, *J* = 7.3 Hz, H-2), 4.68 (1H, d, *J* = 7.3 Hz, H-1), 4.39 (1H, dd, *J* = 7.2, 11.8 Hz, H-6), 2.83 (1H, m, H-5), 2.31 (1H, m, H-11), 2.12 (1H, m, H-7), 1.96 (1H, m, H-8), 1.76 (1H, m, H-9), 1.66 (3H, s, CH_3_-15), 1.54 (1H, m, H-8), 1.48 (1H, m, H-9), 1.21 (3H, s, CH_3_-14), 1.10 (3H, d, *J* = 6.9 Hz, CH_3_-15); ^13^C-NMR (125 MHz, CD_3_COCD_3_) δ: 179.0 (C-12), 142.5 (C-2), 141.8 (C-3), 120.4 (C-1), 114.3 (C-4), 81.1 (C-6), 49.8 (C-5), 43.2 (C-7), 42.4 (C-11), 39.5 (C-10), 34.6 (C-9), 30.7 (C-14), 23.7 (C-8), 22.9 (C-15), 13.0 (C-13).

### Quantification of the Main Components of the EOs

The pure compounds feropodin, (+)-camphor, 1,8-cineole, and β-selinene were dissolved in acetone to make a series of different concentrations (40, 20, 10, 5, 1, and 0.5 μg/ml) and were analyzed using the same GC-MS method as described above. The standard curve for each compound was created on the basis of the concentration and the peak area. Study of the linearity revealed the standard curve equation and correlation coefficient. The standard curve equations of feropodin, (+)-camphor, 1,8-cineole, and β-selinene were *y* = 2.293E-06*x* + 0.3083 (*R*^2^ = 0.9994), *y* = 1.936E-06*x−*0.1727 (*R*^2^ = 0.9998), *y* = 2.672E-07*x* + 0.2027 (*R*^2^ = 0.9992), and *y* = 2.810E-06*x* + 1.826 (*R*^2^ = 0.9991), respectively.

### Fumigant Activity Bioassay

*Spodoptera litura* were raised in the laboratory on an artificial diet, and its eggs were purchased from Keyun Co. (Jiyuan, China). The larvae were reared under controlled photoperiod (light: dark, 16: 8 h) and temperature (24 ± 2°C). Essential oils, (+)-camphor, and 1,8-cineole at each test concentration (80, 60, 40, 20, 10, 5, or 2.5 μl/L) were poured onto a cotton pad and fixed onto the upward-facing side of a triangular flask (Bhavya et al., 2018). The flask was sealed and incubated at 25°C. Ten of the third instar larvae of *S. litura* were placed into each treatment flask, and the treatments were repeated three times, including 240 larvae in total. The *S. litura* larvae were checked every 3 h, and levels of mortality were assessed until 48 h. Control insects were under same conditions without the test compound.

### Antifeedant Activity Bioassay

Following methods previously reported in the literature (Luo et al., 2010; Liu et al., 2021), a serial dilution (1,000, 800, 400, 200, or 100 μg/ml) of each compound was prepared with distilled acetone. Larvae were starved 4 h prior to each bioassay. Fresh leaf disks were cut from *Brassica chinensis*, using a cork borer (0.9 cm in diameter). Leaf disks were smeared either with 10 μl of the distilled acetone solution containing the test compound (treatment group) or with distilled acetone (control group), and repeated this experiment five times. After air drying, two treatment leaf disks and two control ones were set in alternating position in the same Petri dish (9 cm in diameter). Two-thirds of instars were placed at the center of the Petri dish. After feeding for 24 h, areas of leaf disks consumed were measured. The insect antifeedant potency of the test compound was evaluated in terms of the EC_50_ value (the effective dosage for 50% feeding reduction) for each insect species. Commercially available santonin was used as a positive control, which was purchased from Tokyo Chemical Industry Co., Ltd. (TCI, Tokyo, Japan).

### Acetylcholinesterase Inhibition Activity Bioassay

Acetylcholinesterase inhibition activity was evaluated following a detection kit (C1682, Sigma). The test reaction mixture was prepared with phosphate buffer (pH 8.0), crude protein, and the test compound dissolved in DMSO (at a concentration of 100, 50, 30, 10, 3, 1, or 0.2 μg/ml). After incubation for 20 min (37°C), 40 μl of DTNB (0.64 mm) and acetylthiocholine iodide (0.63 mm) were added. Acetylcholinesterase activity was measured at a wavelength of 405 nm every 30 s for 1 h and tacrine was used as positive control. The whole experiment was repeated three times. The AChE inhibition activity was estimated according to the following formula, and by probit analysis to evaluate the IC_50_.

$$\begin{array}{l} \mathrm{Inhibition of AChE}\left(\%\right)\\ =\left[\begin{array}{l} \left(\mathrm{control treatment}-\mathrm{chemical treatment}\right)\\ /\mathrm{control treatment} \end{array}\right]\times 100 \end{array}$$

### Statistical Analysis

The data are expressed as means ± SD of biological replicates. The statistical analyses of the experimental data, including EC_50_, IC_50_, and LC_50_, were conducted using probit run in SPSS 19.0. Where the data of chemical analyses were normally distributed, an independent sample *t*-test was used to compare the measurement data between the two groups, and a Fisher’s and Welch’s one-way ANOVA (Fisher’s and Welch’s one-way ANOVA) was used to compare the measurement data of three or more groups. Differences were considered to be statistically significant, where *p ≤* 0.05.

## Results

### Yield and Compositions of EOs From the Aerial Part of *A. nakaii*

**Figure 1:**
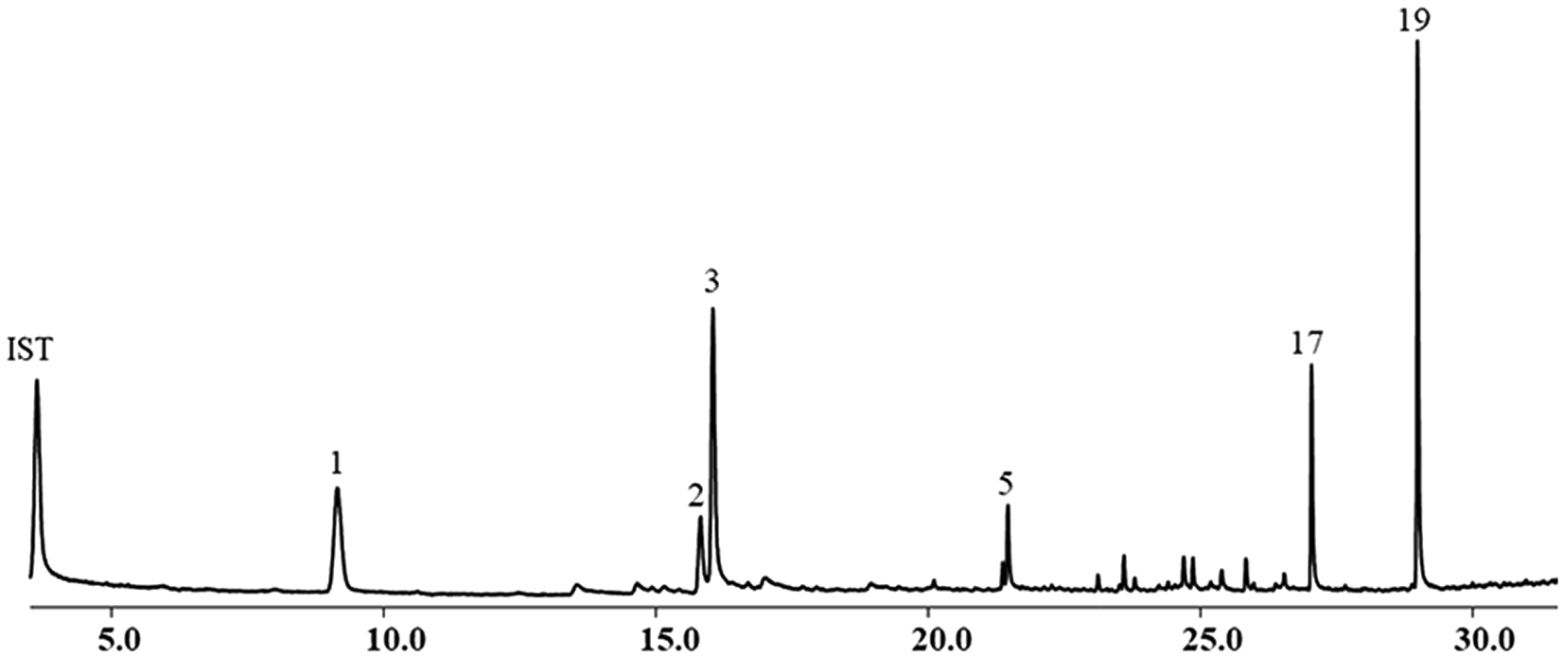
Chemical composition of essential oils (EOs) from *Artemisia nakaii* analyzed using gas chromatograph-mass spectrometer. Nonane was used as an internal standard.

**Figure 2:**
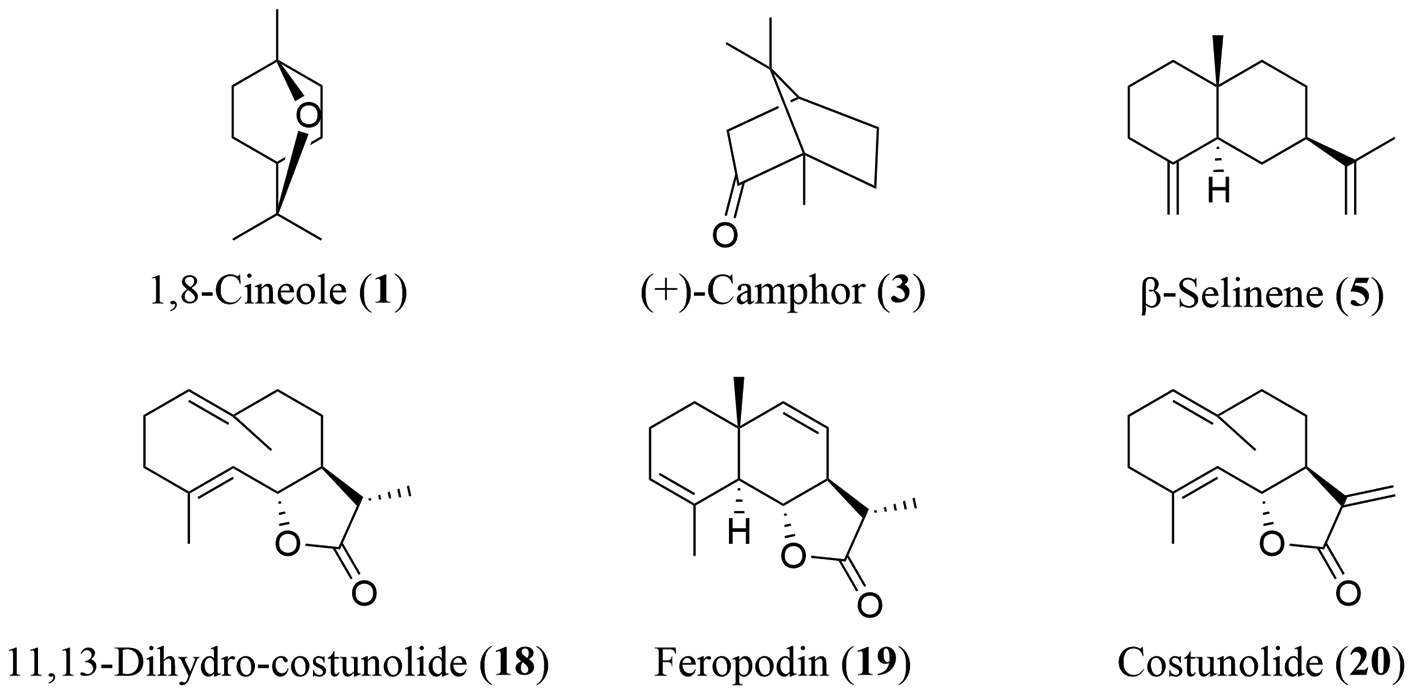
Chemical structures of terpenes in the essential oils extracted from *A. nakaii*.

The yield of EOs from the aerial parts of *A. nakaii* was 0.62 ± 0.07% by hydro-distillation. The GC-MS analyses revealed twenty chromatographic peaks from the EOs (Figure 1). Following comparison with the GC-MS database and known retention indexes, we were able to identify these twenty compounds as being mainly terpenes, including monoterpenes, sesquiterpenes, and their oxygenated derivatives (Table 1). However, the mass spectra of peaks 1 and 3 exhibited 96 and 98% similarity, respectively, with those of the compounds 1,8-cineole and (+)-camphor from the database. The chemical structures of these two compounds were confirmed through comparison of the mass spectra at the retention time with those of the standards. Moreover, peaks 5 and 19 displayed diverse alignment results under the highest similarity being tested at different concentrations. Therefore, in order to elucidate the structures of these two compounds, the EOs were separated on a silica gel column (Sephadex LH-20), followed by semi-preparative HPLC, which yielded four sesquiterpenes. Peaks 5 and 19 structures were then verified as β-selinene and feropodin after comparison with 1D NMR data reported in the literature, and the absolute configuration of feropodin was further determined using 2D NMR spectra (Ando et al., 1991; Chu et al., 2011). Surprisingly, costunolide (peak 20) and 11,13-dihydro-costunolide (peak 18) were segregated in the EOs. These two chemicals are involved in the biosynthetic pathway of feropodin (Figure 2).

**Table 1:** Chemical constitutes of the EOs extracted from *A. nakaii*.

No.	Components	Retention time	Retention index^a^	Retention index^b^	Component in the EOs (%)	Identification^c^
1.	1,8-Cineole	9.15	1,000	1,026	17.77 ± 0.23	S, MS
2.	Borneol	15.83	1,170	1,038	6.82 ± 0.08	MS
3.	(+)-Camphor	16.05	1,177	1,141	24.42 ± 0.65	S, MS
4.	Germacrene D	21.38	1,440	1,484	0.89 ± 0.06	MS
5.	β-Selinene	21.48	1,446	1,489	4.24 ± 0.11	NMR, MS
6.	Isolongifolol	23.13	1,563	1,782	0.62 ± 0.01	MS
7.	Caryophyllene oxide	23.61	1,599	1,582	1.42 ± 0.06	MS
8.	Isocaryophyllene	23.81	1,614	1,408	0.49 ± 0.01	MS
9.	γ-costol	24.26	1,650	1,745	0.31 ± 0.08	MS
10.	8(13)-Dien-5α-ol-4- caryophylla	24.42	1,663	1,639	1.38 ± 0.01	MS
11.	Neointermedeol	24.71	1,687	1,658	1.13 ± 0.21	MS
12.	(+)-Intermedeol	24.88	1,700	1,665	0.24 ± 0.04	MS
13.	α-Selinene	25.41	1,746	1,498	0.92 ± 0.05	MS
14.	4,6,6-Trimethyl-2-(3-methylbuta-1,3-dienyl)-3-oxatricyclo [5.1.0.0(2,4)] octane	25.85	1,784	–	1.31 ± 0.36	MS
15.	*cis*-Lanceol	26.40	1,832	1,760	0.24 ± 0.03	MS
16.	Costol	26.56	1,846	1,773	0.57 ± 1.13	MS
17.	Rishitin	27.06	1,892	–	10.53 ± 0.04	MS
18.	11,13-Dihydro-costunolide	28.54	2,034	–	0.27 ± 0.03	NMR, MS
19.	Feropodin	29.01	2,082	–	26.01 ± 0.74	NMR, MS
20.	Costunolide	29.54	2,136	–	0.19 ± 0.01	NMR, MS

a *Experimentally determined Kováts indices on the SH-RXI-17Sil MS column*.

b *Experimentally determined Kováts indices on the DB-5 column*.

c *MS, mass spectra matched with NIST (2014) data; S, standard; NMR, nuclear magnetic resonance*.

**Figure 3:**
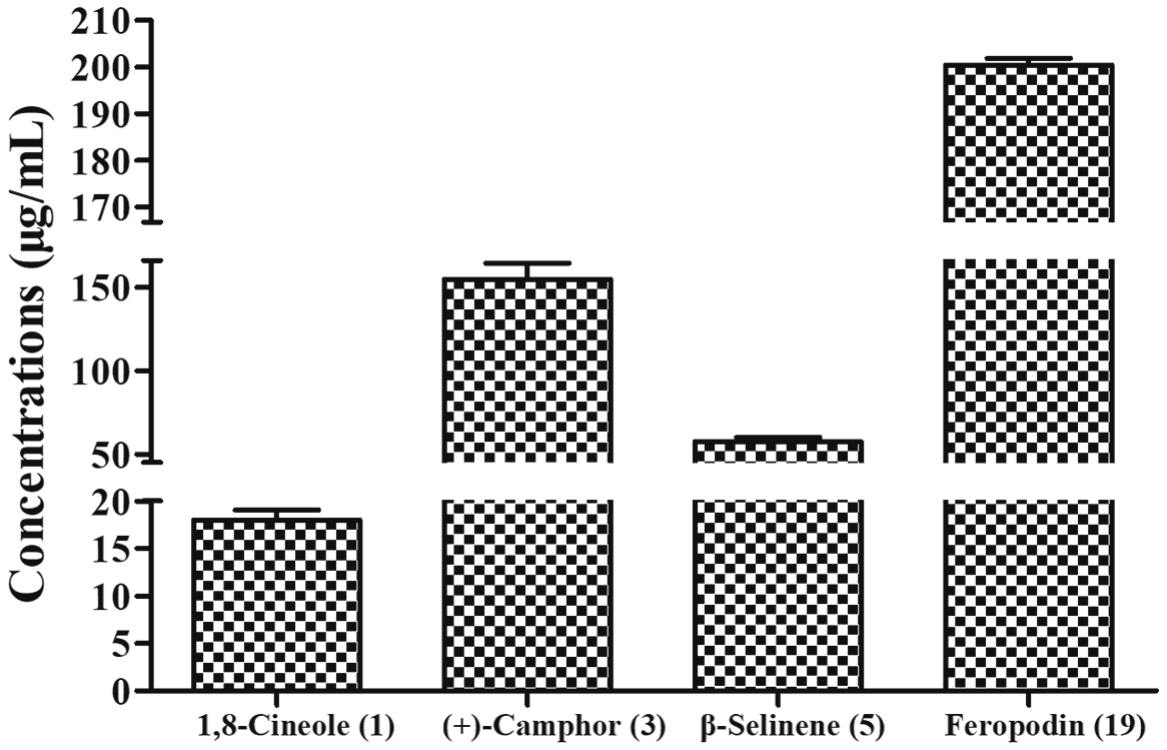
Concentrations of major chemical compounds in *A. nakaii* EOs.

Following area normalization, our results indicated that the dominant monoterpenes in the *A. nakaii* EOs were (+)-camphor (24.42%), 1,8-cineole (17.77%), and borneol (6.82%). Feropodin (26.01%), rishitin (10.53%), and β-selinene (4.24%) accounted for the three most significant sesquiterpenes in the EOs. The EOs were found to contain similar amounts of monoterpene (49.01%) and sesquiterpene (50.76%) hydrocarbons. (+)-Camphor and 1,8-cineole were determined to have concentrations of 154.93 ± 9.72 and 17.99 ± 1.06 μg/ml, respectively, as calculated using external standards. Similarly, the concentration of feropodin in the EOs was found to be 200.46 ± 1.42 μg/ml as calculated using external standards, and β-selinene was at 57.73 ± 2.48 μg/ml (Figure 3).

### Proposed Biosynthetic Pathway of Sesquiterpenes in the EOs of *A. nakaii*

**Figure 4:**
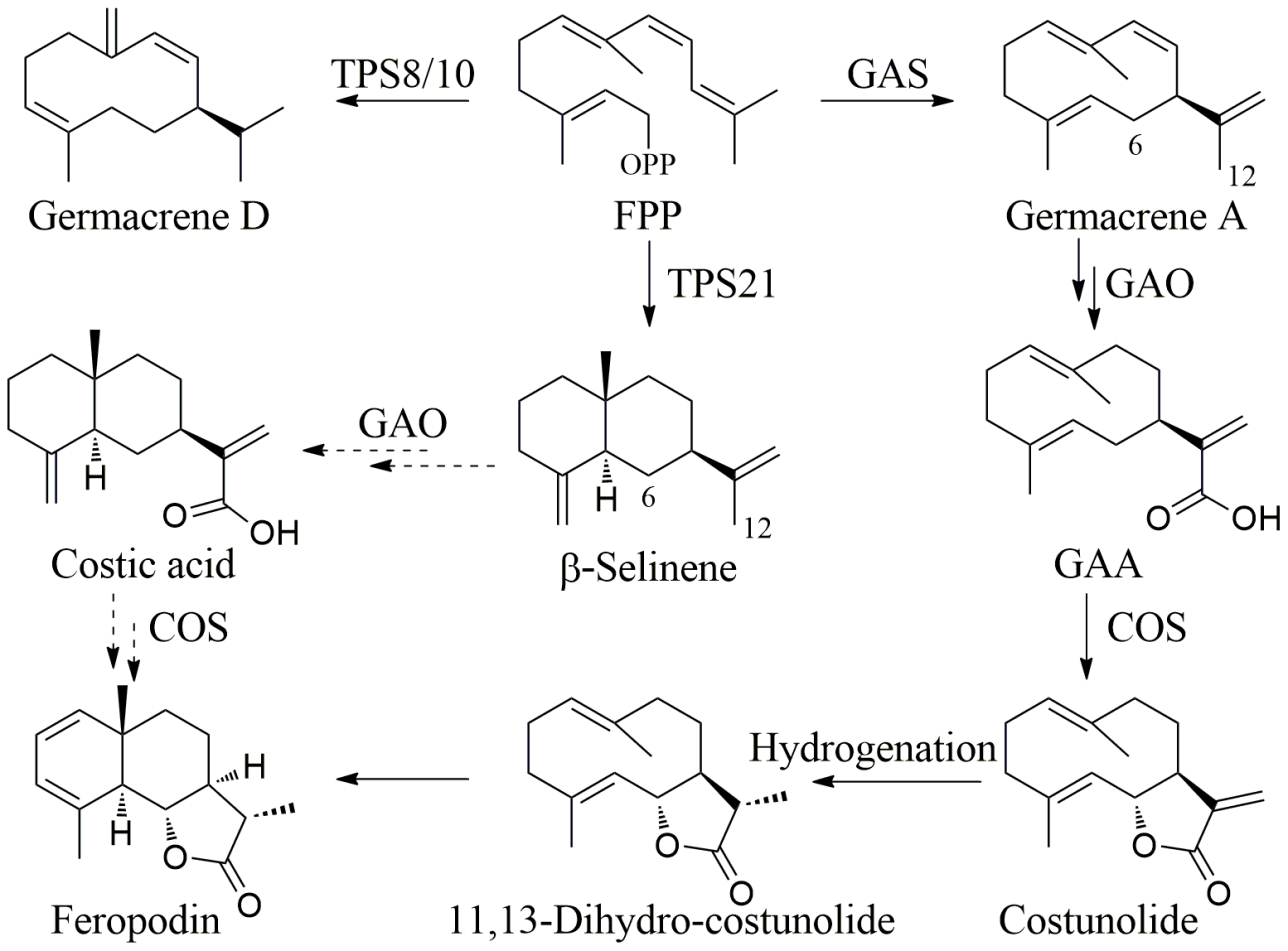
Proposed biosynthetic pathway of sesquiterpenes in the EOs of *A. nakaii*.

The relative percentage of sesquiterpenes in the *A. nakaii* EOs was more than 50%, and feropodin was the sesquiterpene at the highest concentration. Farnesyl diphosphate (FPP) is a substrate of plant sesquiterpene biosynthesis (Liu et al., 2021). The different cyclases GAS, TPS21, or TPS8/10 react with FPP to produce germacrenes, and α- and β-selinenes to produce germacrenes, α-selinene, and β-selinene (Block et al., 2019). The C-12 methyl group of germacrene A is oxidized to germacra-1(10),4,11(13)-trien-12-oic acid (GAA) by germacrene A oxidase, and GAA then undergoes COS-catalyzed 6α-hydroxylation to become costunolide (Ikezawa et al., 2011). Costunolide can be converted to feropodin through the actions of hydrogenase and cyclase. Furthermore, β-selinene can undergo C-12 carboxylation and C-6 hydroxylation through a P450-type reaction, after which spontaneous lactone ring formation can also lead to the formation of feropodin (Figure 4).

### The EOs and Their Monoterpenes Exhibit Fumigant Toxicity Against *S. litura*

**Figure 5:**
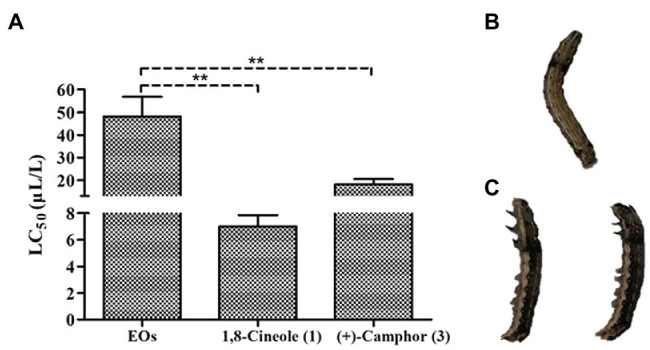
**(A)** Fumigant toxicity of *A. nakaii* EOs and major components against *Spodoptera litura*. **(B)** Fumigant test of control group at 24 h from start of treatment (representative *S. litura* caterpillar). **(C)** Fumigant test of *A. nakaii* EOs under 80 μl/L at 24 h from start of treatment (representative *S. litura* caterpillars). The mean differences were compared using *t*-tests. ^**^ indicates that the value of *p* is less than 0.01.

The EOs extracted from *A. nakaii* exhibited toxicity against third instar larvae of *S. litura*, and the mortality increased with increasing duration of fumigation and concentration of fumigant. Toxicity was seen as early as 6 h at the fumigant concentration of 80 μl/L. Following treatment with EOs fumigant at this concentration for 12 h, some of the larvae had been killed. Following 24 h of treatment, the EOs showed higher toxicity against *S. litura*, with an LC_50_ value of 48.22 ± 8.64 μl/L (Figure 5). Both the major components of the EOs, (+)-camphor and 1,8-cineole, were toxic to third instar larvae of *S. litura*. 1,8-Cineole was acutely lethal, and *S. litura* larvae began to die after only 3 h treatment. 1,8-Cineole showed toxic activity with LC_50_ value of 7.00 ± 0.85 μl/L after 24 h treatment, while (+)-camphor exhibited lower toxicity, with an LC_50_ value of 18.16 ± 2.31 μl/L after 48 h treatment. Therefore, we conclude that both (+)-camphor and 1,8-cineole have insecticidal properties making the EOs of *A. nakaii* to poison *S. litura*.

### The EOs From *A. nakaii* and Their Sesquiterpenes Display Antifeedant Activity Against *S. litura*

**Figure 6:**
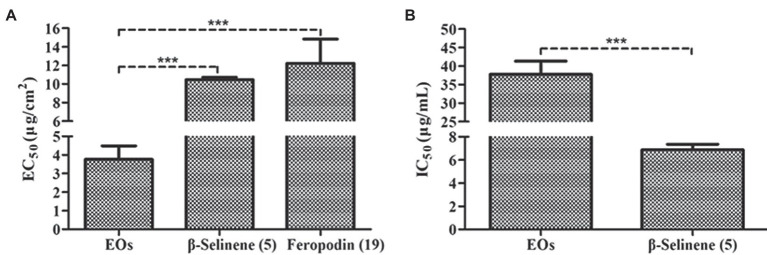
**(A)** Antifeedant activity of *A. nakaii* EOs, β-selinene, and feropodin against *S. litura*. **(B)** Acetylcholinesterase inhibition activity of *A. nakaii* EOs and β-selinene. The mean differences were compared using *t*-tests. ^***^ indicates that the value of *p* is less than 0.001.

Antifeedant activity assays showed that the EOs of *A. nakaii* as well as their main sesquiterpenes (feropodin and β-selinene) exhibited significant antifeedant activity against the larvae of *S. litura*. After 4 h of treatment, control leaf disks without EO treatment experienced significant larval feeding, while treated leaf disks were left nearly intact. The EOs exhibited EC_50_ values of 3.76 ± 0.73 μg/cm^2^. Feropodin was assayed as having EC_50_ values of 12.23 ± 2.60 μg/cm^2^. Furthermore, β-selinene was also found to be a significant deterrent against *S. litura* larvae, with EC_50_ values of 10.46 ± 0.27 μg/cm^2^ (Figure 6A). The positive control, the commercially available santonin, was evaluated as having an antifeedant activity with EC_50_ values of 7.39 ± 1.23 μg/cm^2^.

### The Acetylcholinesterase Inhibition Activity of the EOs and Their Main Components

Acetylcholinesterase is an enzyme critical in regulating nerve transmissions, and numerous of the pesticides induces mortality in pests by disrupting AChE (Bhavya et al., 2018). We wanted to determine whether the insecticidal activity of *A. nakaii* EOs and their major components was related to the activity of AChE. An initial screening was performed to observe AChE inhibition by *A. nakaii* EOs and these compounds, and AChE was found to be strongly inhibited by both the EOs and the major components individually. At a concentration of 10 μg/ml, β-selinene had the strongest AChE inhibition, with a rate of 69.98 ± 2.48%, while feropodin, (+)-camphor, and 1,8-cineole showed lower inhibition, with rates of 5.53 ± 1.88%, 2.23 ± 1.48%, and 11.00 ± 2.09%, respectively. The EOs and β-selinene showed IC_50_ values of 37.75 ± 3.59 and 6.88 ± 0.48 μg/ml, respectively (Figure 6B). The IC_50_ value of the positive control (tacrine) was 0.026 ± 0.0012 μg/ml.

## Discussion

*Artemisia* is belongs to the Asteraceae family and comprised of about 500 species, tending to be rich in EOs (Abad et al., 2012). The EOs of *Artemisia* genus plants have been researched to display insecticidal activities against a wild range of pests (Islamuddin et al., 2014; Gao et al., 2020). Fumigant and contact toxicities of EOs from *A. herba-alba* and *A. absinthium* have been studied toward *Orysaephilus surinamensis* and *T. castaneum*, and they are potential to exploit into an inartificial contact insecticide (Bachrouch et al., 2015). The EOs from *A. sieberi* were researched possessing fumigant and sublethal activities against *Sitotroga cerealella* (Naseri et al., 2017). The EOs from *A. absinthium* have been discovered as an existing nematicidal activity against *Meloidogyne incognita* (Kundu et al., 2020). The EOs from *A. campestris* showed toxicity on *Culex quinquefasciatus* (Ammar et al., 2020). Coincidentally, we found that the EOs isolated from the aerial parts of *A. nakaii* had high yield and exhibited fumigant toxicity against the tobacco cutworm moth (*S. litura*). Hence, the EOs from *A. nakaii* and their various and active ingredients are worth particular attention.

EOs are often complex mixtures of chemicals, commonly containing terpenes, phenolics, and fatty acids (Passreiter et al., 2004; Toscano-Garibay et al., 2017). The EOs of *Tanacetum balsamita* L. were found to contain β-thujone (84.43%), α-thujone (4.68%), eucalyptol (4.07%), and aliphatics (1.82%). Our previous studies have shown terpenes to be the major components of the EOs of *Thuja occidentalis* (Bai et al., 2020). The EOs obtained from *A. nakaii* comprised mostly monoterpenes and sesquiterpenes, including feropodin (26.01%), (+)-camphor (24.42%), 1,8-cineole (17.77%), rishitin (10.53%), borneol (6.82%), and β-selinene (4.24%). Furthermore, two of the precursors involved in the biosynthetic pathway of feropodin, costunolide, and 11,13-dihydro-costunolide were found separately in the EOs from *A. nakaii*.

Current *S. litura* control programs rely heavily on chemical insecticides, but most of these have failed to adequately control the insects (Vasantha-Srinivasan et al., 2016). Pesticides not only have high economic and environmental costs, but also have resulted in the appearance of insecticide-resistant pest populations (Baccari et al., 2020). Plant EOs may provide eco-friendly compounds suitable for using as conventional insecticides due to their minimal side effects on the environment and human health (Gonzalez-Mas et al., 2019). Meanwhile, our studies revealed that (+)-camphor and 1,8-cineole both have pesticidal activity against *S. litura* individually, and the effects are stronger than that of the EOs. Accordingly, (+)-camphor and 1,8-cineole may be contributing to the toxicity of *A. nakaii* EOs. Moreover, the capacity of Asteraceae EOs as botanical pesticides for the use in tomato production has been demonstrated. Essential oils were extracted from the vegetative growth stage of *A. annua* having the potential to control pest (Oftadeh et al., 2020). Therefore, *A. nakaii* EOs or its components may become the potential alternatives to chemical pesticides, effective in the control of *S. litura* larvae.

Terpenoid compounds acquired from EOs reveal various insecticidal activities and complex mechanisms of action. The insecticidal activity of EOs, monoterpenes and sesquiterpenes, such as carvacrol, is often correlated with their ability to inhibit AChE (Yeom et al., 2012). Much research has demonstrated that there is a connection between the insecticidal activity of β-phellandrene and its ability to inhibit AChE. We determined the AChE inhibition of the EOs extracted from *A. nakaii*, as well as their major components, and found that the highest AChE inhibition was shown by the EOs and β-selinene, with values of 37.75 ± 3.59 and 6.88 ± 0.48 μg/ml, respectively. In our experiments, feropodin, (+)-camphor, and 1,8-cineole were ineffective against AChE. This is consistent with our antifeedant activities results, and AChE deactivation in *S. litura* could therefore be the mechanism of action of the antifeedants in *A. nakaii* EOs and β-selinene. However, 1,8-cineole has been reported previously as having AChE inhibitory activity (Dohi et al., 2009), indicating the various actions related to the major component 1,8-cineole. Consequently, the insecticidal toxicities of *A. nakaii* EOs appear to be related to its main constituents of 1,8-cineole, (+)-camphor, and β-selinene.

## Conclusion

In this study, twenty terpenes were identified from the EOs of *A. nakaii*, with feropodin (+)-camphor, 1,8-cineole, rishitin, borneol, and β-selinene being the major constituents. Using the external standard methods, we were able to precisely quantify of the EO components feropodin, (+)-camphor, 1,8-cineole, and β-selinene in the EOs. Antifeedant activity of *A. nakaii* EOs, β-selinene, and feropodin was evident against *S. litura*. The EOs, (+)-camphor, and 1,8-cineole showed pronounced fumigant activity against third instar larvae of *S. litura*, which became more obvious at high concentrations. Furthermore, both 1,8-cineole and (+)-camphor alone had better insecticidal activity than the EOs. Thus, 1,8-cineole and (+)-camphor might be the substances responsible for the EOs activity against *S. litura*. In addition, both the EOs and β-selinene alone inhibited AChE activity, which is a possible mechanism of action of the observed antifeedant activity. Hence, the terpenes from EOs of *A. nakaii* may be regarded as good candidates for new botanical insecticides to reduce the use of conventional pesticides in the control of *S. litura*.

## Data Availability Statement

The datasets presented in this study can be found in online repositories. The names of the repository/repositories and accession number(s) can be found in the article/Supplementary Material.

## Author Contributions

JL, JH, BQ, MS, XG, YW, and SL designed the research and performed the experiments. JL, JH, MS, and BQ analyzed the data. SL and JL wrote the paper and conceived the project. All authors contributed to the article and approved the submitted version.

## Conflict of Interest

The authors declare that the research was conducted in the absence of any commercial or financial relationships that could be construed as a potential conflict of interest.

## Publisher’s Note

All claims expressed in this article are solely those of the authors and do not necessarily represent those of their affiliated organizations, or those of the publisher, the editors and the reviewers. Any product that may be evaluated in this article, or claim that may be made by its manufacturer, is not guaranteed or endorsed by the publisher.

## Supplementary Material

The Supplementary Material for this article can be found online at Figshare via the DOI 10.6084/m9.figshare.14730639.v1
